# Impact of CD56 Continuously Recognizable as Prognostic Value of Acute Promyelocytic Leukemia: Results of Multivariate Analyses in the Japan Adult Leukemia Study Group (JALSG)-APL204 Study and a Review of the Literature

**DOI:** 10.3390/cancers12061444

**Published:** 2020-06-01

**Authors:** Akihiro Takeshita, Norio Asou, Yoshiko Atsuta, Hiroaki Furumaki, Toru Sakura, Yasunori Ueda, Masashi Sawa, Nobuaki Dobashi, Yasuhiro Taniguchi, Rikio Suzuki, Masaru Nakagawa, Shigehisa Tamaki, Maki Hagihara, Katsumichi Fujimaki, Hitoshi Minamiguchi, Hiroyuki Fujita, Masamitsu Yanada, Yoshinobu Maeda, Noriko Usui, Yukio Kobayashi, Hitoshi Kiyoi, Shigeki Ohtake, Itaru Matsumura, Tomoki Naoe, Yasushi Miyazaki, the Japan Adult Leukemia Study Group

**Affiliations:** 1 Transfusion and Cell Therapy, Hamamatsu University School of Medicine, 1-20-1 Handayama, Hamamatsu, Higashiku 431-3192, Japan; hirofuru@hama-med.ac.jp; 2 International Medical Center, Saitama Medical University, 1397-1, Yamane, Hidaka 350-1298, Japan; ktcnasou@saitama-med.ac.jp; 3 The Japanese Data Center for Hematopoietic Cell Transplantation, 1-1-20 Taikou-minami, Higashiku, Nagoya 461-0047, Japan; y-atsuta@jdchct.or.jp; 4 Hematology, Saiseikai Maebashi Hospital, 564-1, Kamishindenmachi, Maebashi 371-0821, Japan; tor-sakura@maebashi.saiseikai.or.jp; 5 Hematology/Oncology, Kurashiki Central Hospital, 1-1-1 Miwa, Kurashiki 710-8602, Japan; ueda-y@kchnet.or.jp; 6 Hematology and Oncology, Anjo Kosei Hospital, 28 Higashikurokute, Anjochou, Anjo 446-8602, Japan; msawa@wine.plala.or.jp; 7 Clinical Oncology/Hematology, Department of Internal Medicine, The Jikei University School of Medicine, 3-25-8, Nishisinbashi, Minatoku, Tokyo 105-8461, Japan; dobashi@jikei.ac.jp (N.D.); usuin@jikei.ac.jp (N.U.); 8 Hematology and Rheumatology, Kindai University Faculty of Medicine, 377-2 Ohnohigashi, Ohsakasayama 589-8511, Japan; m11049@med.kindai.ac.jp (Y.T.); imatsumura@med.kindai.ac.jp (I.M.); 9 Hematology and Oncology, Tokai University School of Medicine, 143 Shimokasuya, Isahara 259-1193, Japan; Rikio_Suzuki@tsc.u-tokai.ac.jp; 10 Hematology and Rheumatology, Nihon University School of Medicine, 30-1 Ohyaguchikamichou, Itabashiku, Tokyo 173-8610, Japan; nakagawa.masaru@nihon-u.ac.jp; 11 Hematology, Japanese Red Cross Ise Hospital, 1-471-2 Funae, Ise 516-8512, Japan; stamaki@ise.jrc.or.jp; 12 Hematology and Clinical Immunology, Yokohama City University School of Medicine, 3-9 Fukuura, Kanazawaku, Yokohama 236-0004, Japan; makigon@yokohama-cu.ac.jp; 13 Hematology, Fujisawa City Hospital, 2-6-1 Fujisawa, Fujisawa 251-8550, Japan; kkfujimaki@gmail.com; 14 Hematology, Shiga University of Medical Science, Seta-Tsukinowa, Otsu 520-2192, Japan; minamigh@belle.shiga-med.ac.jp; 15 Hematology, Saiseikai Yokohama Nanbu Hospital, 3-2-10 Kounandai, Kounanku, Yokohama 234-0054, Japan; fujitah@nanbu.saiseikai.or.jp; 16 Hematology and Cell Therapy, Aichi Cancer Center, 1-1 Kanokoden, Chikusaku, Nagoya 464-8681, Japan; myanada@aichi-cc.jp; 17 Hematology, Oncology and Respiratory Medicine, Okayama University Medical School, 2-5-1 Shikatachou, Kitaku, Okayama 700-8558, Japan; yosmaeda@md.okayama-u.ac.jp; 18 National Cancer Center Hospital, 5-1-1 Tsukiji, Chuouku, Tokyo 104-0045, Japan; ykkobaya@iuhw.ac.jp; 19 Hematology and Oncology, Nagoya University Graduate School of Medicine, 65 Tsurumaichou, Showaku, Nagoya 466-8550, Japan; kiyoi@med.nagoya-u.ac.jp; 20 Kanazawa University, Kakumamachi, Kanazawa 920-1192, Japan; sohtake@staff.kanazawa-u.ac.jp; 21 National Hospital Organization Nagoya Medical Center, 4-1-1 Sannomaru, Nakaku, Nagoya 460-0001, Japan; naoe.tomoki.wx@mail.hosp.go.jp; 22 Hematology, Atomic Bomb Institute, Nagasaki University, 1-12-4 Sakamoto, Nagasaki 852-8523, Japan; y-miyaza@nagasaki-u.ac.jp; 23 JALSG office, 3-6-35 Nishiki, Nakaku, Nagoya 460-0003, Japan; office@jalsg.jp

**Keywords:** acute promyelocytic leukemia, prognosis, multivariate analysis, tamibarotene, CD56

## Abstract

Background: After long-term analysis of the JALSG-APL204 study we recently reported that maintenance therapy with tamibarotene was more effective than all-*trans* retinoic acid (ATRA) by reducing relapse in APL patients. Here, the clinical significance of other important prognostic factors was evaluated with multivariate analyses. Patients and Methods: Newly diagnosed acute promyelocytic leukemia (APL) patients were registered with the study. Induction was composed of ATRA and chemotherapy. Patients who achieved molecular remission after consolidation were randomly assigned to maintenance with tamibarotene or ATRA. Results: Of the 344 eligible patients, 319 (93%) achieved complete remission (CR). After completing consolidation, 269 patients underwent maintenance random assignment—135 to ATRA, and 134 to tamibarotene. By multivariate analysis, overexpression of CD56 in blast was an independent unfavorable prognostic factor for relapse-free survival (RFS) (*p* = 0.006) together with more than 10.0 × 10^9^/L WBC counts (*p* = 0.001) and the ATRA arm in maintenance (*p* = 0.028). Of all phenotypes, CD56 was related most clearly to an unfavorable prognosis. The CR rate, mortality rate during induction and overall survival of CD56^+^ APL were not significantly different compared with CD56^−^ APL. CD56 is continuously an independent unfavorable prognostic factor for RFS in APL patients treated with ATRA and chemotherapy followed by ATRA or tamibarotene maintenance therapy.

## 1. Introduction

The treatment outcome of acute promyelocytic leukemia (APL) has markedly improved over the past three decades following the development of novel agents including all-*trans* retinoic acid (ATRA), arsenic trioxide (ATO) and chemotherapy [1,2,3,4,5,6,7]. Recently, 90% of patients with APL achieve complete remission (CR) after induction therapy, and 80% of patients maintain long-term, disease-free survival. However, several % of patients in the low-risk group and 10–20% of those in the high-risk group have a recurrence of the disease after the first remission [8,9,10,11,12,13]. Treatment of patients in the high-risk group for APL has therefore been a major focus of attention in this area. Analyses of prognostic factors is still crucial in the management of APL.

Various prognostic factors with an expected outcome have been reported. Specifically, high white blood cell (WBC) count with or without low platelet count before the induction treatment have been recognized as significant factors [7,9,10,11,12]. More detailed analyses have shown the relationship between a poor outcome and several characteristics, including older age, chromosomal abnormalities other than t (15;17), phenotypic features, FLT3 mutations and presence of the *PML-RARA* isoform [13,14,15,16,17]. However, these observations have not received approval to amend the standard therapy for APL [18,19,20].

Recently, we analyzed the long-term outcomes of the Japan Adult Leukemia Study Group (JALSG) APL 204 study, prospectively treated with ATRA combined with chemotherapies followed by maintenance therapy with ATRA or tamibarotene [21,22]. Tamibarotene, a synthetic retinoid, is chemically more stable to light, heat and oxidation than ATRA, and is approximately 10 times more potent in its ability to induce in vitro differentiation [23,24]. Tamibarotene displays a low affinity for cellular retinoic acid binding protein, the overexpression of which is associated with ATRA resistance. Moreover, unlike ATRA, the plasma level of tamibarotene does not decline after daily administration. We have shown that tamibarotene is superior to ATRA by decreasing the incidence of relapse [21,22,25,26]. Additionally, we showed that a high WBC count at diagnosis is one of the significant prognostic factors for poor relapse-free survival (RFS) [22]. Here, we precisely analyzed the data of the APL204 study at a median follow-up of 7.3 years. Our aim was to identify important prognostic factors in 344 APL patients enrolled in the study, of which 269 underwent maintenance randomization. Moreover, we compared these patients with 302 patients enrolled in our previous APL97 study (a median follow-up of 8.5 years) who underwent ATRA treatment and chemotherapy with or without intensive maintenance chemotherapy [7,27].

## 2. Materials and Methods

### 2.1. Patients

Adult patients with previously untreated APL with t (15;17) and/or the *PML-RARA* were enrolled onto the JALSG-APL204 study between April 2004 and December 2011 [21,22]. Other eligibility criteria included age between 15 and 70 years, Eastern Cooperative Oncology Group (ECOG) performance status (PS) 0 to 3, and sufficient functioning of the heart, lung, liver and kidney. Written informed consent was obtained from each patient before registration to the study in accordance with the Declaration of Helsinki. This study was approved by the institutional review boards of each participating institution and registered at the University Hospital Medical Information Network Clinical Trials Registry under C000000154.

### 2.2. Treatments

**Figure 1:**
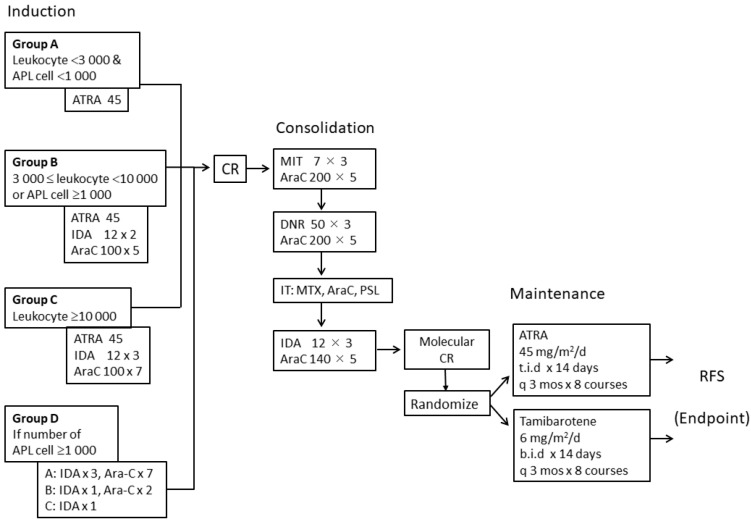
Scheme of the APL204 study. AraC, cytarabine; ATRA, all-*trans* retinoic acid; DNR, daunorubicin; IDA, idarubicin; MIT, mitoxantrone; MTX, methotrexate; PSL, prednisolone; IT, intrathecal injection; CR, complete remission; RFS, relapse-free survival.

The JALSG-APL204 is a randomized controlled, phase three multicenter study [21]. An outline of the treatment schedule is reproduced in Figure 1. [22] For remission induction therapy, ATRA (45 mg/m^2^/day) was given until complete remission (CR) for up to 60 days. In accordance with previous JALSG APL studies, simultaneous chemotherapy with idarubicin (IDA) and cytarabine (Ara-C) was given in accordance with the initial WBC and blast count in the peripheral blood [7]. After achieving complete remission (CR), three courses of intensive consolidation chemotherapy including anthracyclines and Ara-C were given; in particular, mitoxantrone 7 mg/m^2^ on days one to three and Ara-C 200 mg/m^2^ on days one to five for the first course; daunorubicin 50 mg/m^2^ on days one to three and Ara-C 200 mg/m^2^ on days one to five for the second course; and IDA 12 mg/m^2^ on days one to three and Ara-C 140 mg/m^2^ on days one to five for the third course. Intrathecal injection (IT) was given after recovery from the second consolidation course as prophylaxis for central nervous system (CNS) leukemia. Patients, whose *PML-RARA* fusion transcripts were not found after consolidation, were randomly allocated either to ATRA (45 mg/m^2^/day) or tamibarotene (6 mg/m^2^/day) maintenance for 14 days every three months for up to two years.

CR and hematological relapse were defined to be consistent with previous reports [7,21]. *PML-RARA* transcript levels were evaluated in bone barrow after recovery of the third consolidation therapy, and then after every two courses of maintenance therapy, and every six months thereafter. Transcript levels were determined using the real-time quantitative reverse transcription polymerase chain reaction (RQ-PCR) assay [7,21]. Molecular remission was defined by *PML-RARA* transcript levels as being less than 100 copies/μg RNA. Molecular relapse was defined as a loss of molecular remission confirmed in two consecutive bone marrow samples taken at one-month intervals.

### 2.3. Immunophenotypic Analyses

Immunophenotypic analyses were performed using bone marrow samples at diagnosis by flow cytometry. Cells were stained with anti-CD45 monoclonal antibody (mAb), gated by CD45 expression and side scatter (SSC), and analyzed by fluorescein conjugated mAb against CD2, CD5, CD7, CD4, CD8, CD19, CD20, CD11b, CD13, CD14, CD15, CD33, CD34, CD56 and HLA-DR antigens. In accordance with the EGIL criteria [28], surface markers generally not determined on APL cells were defined as positive if more than 10% of APL cells expressed the corresponding antigens.

### 2.4. Definition of Outcomes

Relapse-free survival (RFS) was defined as the time from random assignment to hematological or molecular relapse, death or last visit, whichever came first. Overall survival (OS), event-free survival (EFS), cumulative incidence of relapse (CIR), RFS in the initial treatment groups and RFS in risk groups were also analyzed using standard definitions as described in our previous paper [7].

### 2.5. Statistical Analysis

Long-term survival, disease status and late complications at 7.3 years were collected between January 2016 and June 2018. Categorical data were compared using χ^2^-test and Fisher’s exact test for categorical variables and Wilcoxon rank sum test for continuous variables. The probabilities of RFS, OS and EFS were estimated using the Kaplan–Meier method. CIR was analyzed by Gray’s test [29]. The Cox proportional hazards regression model was used for calculating the hazard ratio (HR) in conjunction with the 95% confidence interval (CI). Factors significant at the 0.2 level in the univariate analysis were included in the multivariate analysis model. Statistical analyses were performed using SPSS 25.0 (SPSS Inc, Chicago, IL, USA) and EZR 1.37, a graphical user interface for the R software program (The R Foundation for Statistical Computing, Vienna, Austria). All hypothesis testing was two-tailed with a significance level of 0.05.

## 3. Results

### 3.1. Patient Characteristics

Between April 2004 and December 2010, 347 newly diagnosed patients with APL were enrolled for this study, of which 344 were eligible for analysis [21,22]. The median follow-up period was 7.3 years (0 to 12.3 years). Table 1 shows the baseline characteristics of the eligible patients. Among them, 325 (94%) (median age, 48 years; range, 15 to 70) had satisfactory data of the CD phenotype and were evaluated in this study.

### 3.2. Treatment Outcome

**Figure 2:**
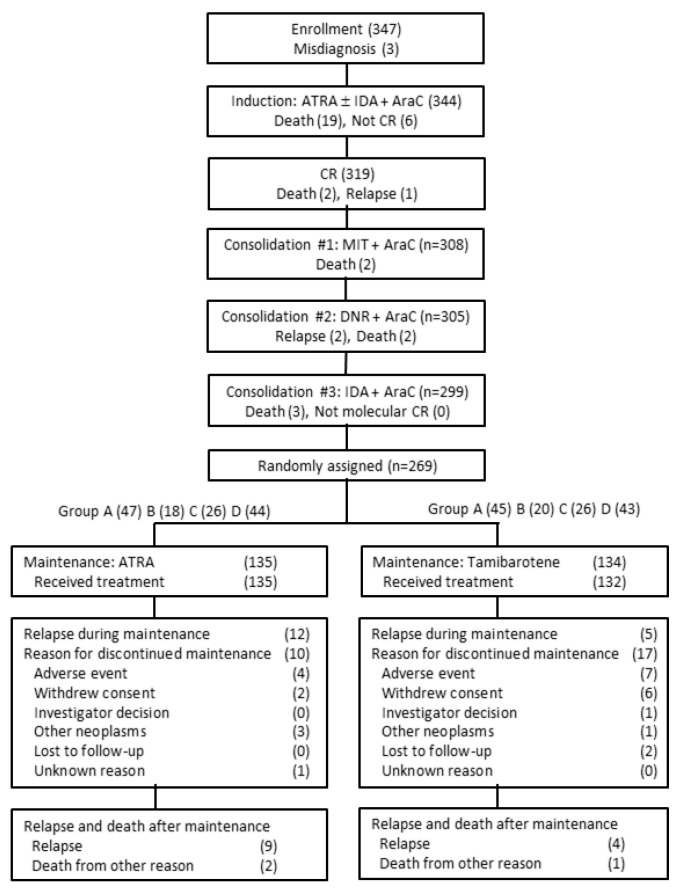
A CONSORT diagram before and after random assignment of the APL204 study. Numbers in parentheses refer to the numbers of patients. ATRA, all-*trans* retinoic acid; DNR, daunorubicin; IDA, idarubicin; MIT, mitoxantrone; AraC, cytarabine; CR, complete remission.

Of the 344 eligible patients, 319 (93%) achieved CR. After completing consolidation chemotherapy, 269 patients underwent maintenance random assignment; 135 were given ATRA, and 134 were given tamibarotene. A CONSORT diagram is summarized and reproduced in Figure 2. [22] Results from univariate analysis of risk factors for CR are given in Table 2. Patients with initial WBC counts of 3.0 × 10^9^/L or more had a lower CR rate compared to those with initial WBC counts of less than 3.0 × 10^9^/L (*p* = 0.011). Overexpression of CD phenotypes CD34 and CD56 in relation to CR rate were also analyzed (*p* = 0.417 and *p* = 0.212, respectively). Death within 30 days was compared with clinical features and reported elsewhere. In brief, the mortality rate increased in patients with initial WBC counts of 3.0 × 10^9^/L or more (*p* = 0.002), platelet counts of less than 40.0 × 10^9^/L (*p* = 0.026) and those with variant FAB subtype (*p* = 0.031) and a higher Sanz score (*p* = 0.008). Three of 344 patients had refractoriness to the induction. The incidence of differentiation syndrome did not relate to any of the clinical features.

Table 3a summarizes the results from univariate analysis on RFS, which is the primary endpoint of this study. Univariate regression analysis found several risk factors for adverse prognosis including initial WBC count (≥10.0 × 10^9^/L) (*p* < 0.001), Sanz score (*p* = 0.001), CD34^+^ (*p* = 0.040), CD56^+^ blast (≥10%) (*p* = 0.005) and the ATRA arm in maintenance therapy (*p* = 0.027). By contrast, age, sex, PS, and chromosome abnormality other than t (15; 17) were not significant factors. The unique factors found to be significant in univariate regression analysis were included in the multivariate analysis of risk factors for adverse prognosis (Table 3b). Consequently, overexpression of CD56 in blast was an independent unfavorable prognostic factor for RFS (HR = 3.19, 95% CI 1.40–7.27, *p* = 0.006) together with a WBC count of more than 10.0 × 10^9^/L (*p* = 0.001) and the ATRA arm in maintenance therapy (*p* = 0.028). The latter two factors were reported in our previous report on the primary endpoint [21,22]. The relationships between CD phenotypes and clinical outcomes are summarized in Table 4. Of all CD phenotypes, CD56 was related most clearly to adverse prognosis. Therefore, we focused on the clinical impact of CD56 on treatment outcome in relation to other prognostic factors.

Of the 325 patients that were analyzable, 45 (13.8%) were positive for CD56. The clinical and biological characteristics according to CD56 expression are shown in Table 5. CD56 expression was not related to any of these characteristics. As for the relationship of CD56 with other CD phenotypes, a significant correlation was found with each of CD2, CD7, CD34, HLA-DR (*p* < 0.001, each), but not with each of CD11b and CD15 (*p* = 0.096 and *p* = 0.339, respectively). However, none of these except CD56 related to clinical outcome in the multivariate analysis.

**Figure 3:**
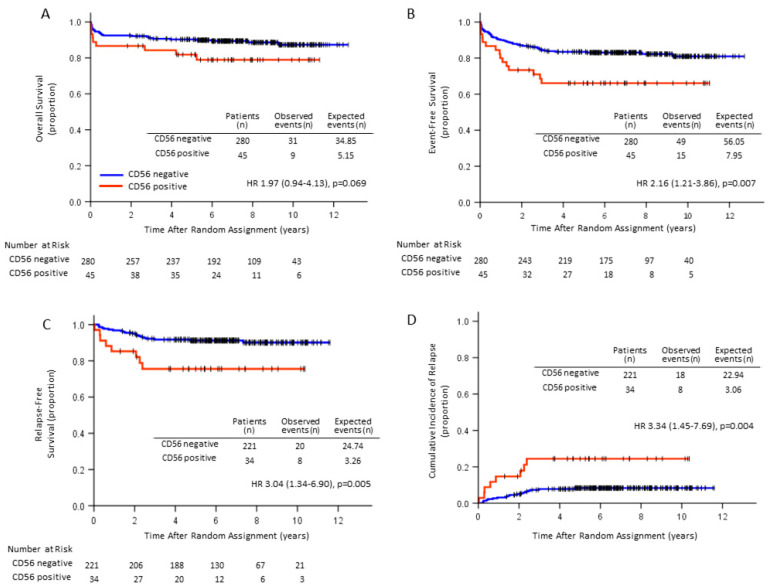
Long-term Kaplan–Meier curves of OS (**A**), EFS (**B**), RFS (**C**) and CIR (**D**) according to CD56 expression. EFS, RFS and CIR were inferior in CD56^+^ APL (*p* = 0.007, *p* = 0.005, *p* = 0.004, respectively) than CD56^−^ APL, while OS was not significantly different between the two groups (*p* = 0.069).

**Figure 4:**
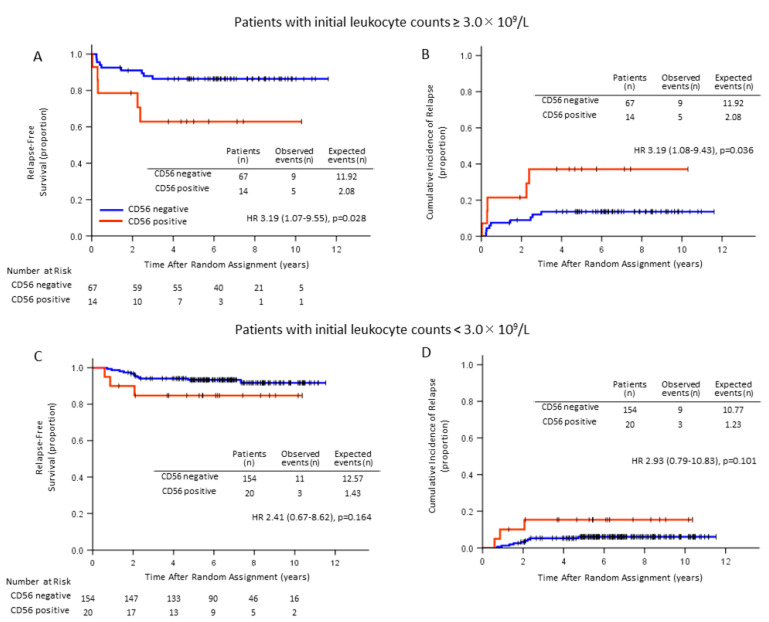
RFS and CIR according to CD56 expression and initial leukocyte count. RFS (**A**) and CIR (**B**) patients with an initial leukocyte count of ≥3.0 × 10^9^/L (*n* = 81) were significantly different between CD56^+^ and CD56^−^ patients (*p* = 0.028 and *p* = 0.036, respectively). However, CD56^+^ and CD56^−^ patients (**C** and **D**, respectively) with an initial leukocyte count of <3.0 × 10^9^/L (*n* = 174) showed no significant difference (*p* = 0.164 and *p* = 0.101, respectively).

EFS, RFS and CIR were inferior in CD56^+^ APL (66.1% vs. 83.1%, *p* = 0.007, 76.5% vs. 91.4%, *p* = 0.005, HR 3.04 (1.34–6.90) and 23.5% vs. 8.1%, *p* = 0.004, HR 3.34 (1.45–7.69, respectively) than for CD56^−^ APL, while OS was not significantly different between the two groups (78.9% vs. 89.4%, *p* = 0.069) (Figure 3). In patients with initial WBC counts of 3.0 × 10^9^/L or more, RFS and CIR for 14 CD56^+^APL patients were significantly inferior to those for 67 CD56^−^APL patients (64.3% vs. 86.6%, *p* = 0.028, and 35.7% vs. 13.4%, *p* = 0.036, respectively; Figure 4), while in patients with initial WBC counts of less than 3.0 × 10^9^/L, RFS and CIR were not significantly different between the two groups (*p* = 0.164 and *p* = 0.101, respectively). In a limited number of patients, OS was not significantly different between the two groups regardless of the initial WBC count. RFS and CIR for 8 CD56^+^APL patients among those with initial WBC counts of 10.0 × 10^9^/L or more were not significantly different from those for 43 CD56^−^APL patients (62.5% vs. 79.1%, *p* = 0.200, and 20.9% vs. 37.5%, *p* = 0.220, respectively). We also analyzed the influence of CD56 expression on clinical outcomes according to Sanz’s relapse-risk score [4]. OS, EFS, RFS, and CIR were not significantly different between CD56^−^ and CD56^+^ patients in the high-risk group. Among 221 CD56^−^ patients, RFS in patients treated with tamibarotene was significantly better than that with ATRA (*p* = 0.001), but not in 34 CD56^+^ patients (*p* = 0.359). These observations might be explained by the small number of CD56^+^ cases in the high-risk group. Therefore, we analyzed the differences in the high- and intermediate-risk groups together. The RFS and CIR were significantly inferior in 26 CD56^+^ patients (76.5% vs. 90.4%, *p* = 0.039 and 23.1% vs. 9.5%, *p* = 0.037, respectively), while OS and EFS were unchanged (*p* = 0.202 and *p* = 0.082).

In addition, we analyzed the outcome of CD34^−^CD56^−^ (177 cases), CD34^+^CD56^−^ (44 cases), CD34^−^CD56^+^ (13 cases) and CD34^+^CD56^+^ (21 cases) groups. RFS of these were 92.5%, 85.9%, 76.9% and 75.6%, respectively. (CD34^−^CD56^−^ vs. CD34^+^CD56^−^, *p* = 0.083; CD34^−^CD56^−^ vs. CD34^−^CD56^+^, *p* = 0.019; and CD34^−^CD56^−^ vs. CD34^+^CD56^+^, *p* = 0.010).

## 4. Discussion

We recently reported that tamibarotene maintenance improved RFS of APL in our JALSG-APL204 study with a median follow-up of 7.3 years (HR = 0.44, 95%CI 0.21-0.93, *p* = 0.027) [22]. This observation was more pronounced in high-risk patients with an initial leukocyte count of ≥10.0 × 10^9^/L (HR = 0.27, 0.07–0.99, *p* = 0.034). We further evaluated other important prognostic factors with multivariate analysis. In particular, immunophenotypes were extracted during this evaluation.

The relationships between immunophenotypes and clinical outcome have been reported in AML. Of all immunophenotypes, overexpression of CD56 has been reported in 15% to 20% of AML patients with poorer survival [30,31]. This observation has been reported in several AML subtypes having *RUNX1-RUNX1T1* or *PML-RARA* [14,30,31] and is thought to be related to hyperleukocytosis or extramedullary involvement [32,33,34]. These findings indicate that CD56 is related to the progression of AML and resistance to therapy.

Previous reports have suggested that overexpression of CD2, CD34, HLA-DR and CD56 in APL patients is associated with poorer clinical outcomes [14,27,35,36,37,38]. Our study indicates CD2, CD7, CD34 and CD56 are associated with a poorer clinical outcome, and CD56 was extracted in multivariate analysis. CD56 is expressed in around 10% of patients with APL [39,40,41,42]. Our previous report on long-term survival of APL97, which analyzed 239 patients with APL, also highlighted the prognostic significance of CD56 expression [27]. The study showed CD56 expression was correlated with lower platelet counts and severe intravascular coagulation before induction therapy, but not with higher WBC counts, lower albumin levels and higher frequency of M3 variant, as reported previously [39,41]. Indeed, in the analysis of APL204, overexpression of CD56 was not correlated with any of these clinical features before induction therapy. This observation suggests that long-term outcomes in APL204 were improved by comparison to those in APL97. Prognostic factors are often difficult to extract in a developed regimen.

There was no difference in each of CR and induction mortality between the CD56^+^ and CD56^−^ groups in our study [27]. The PETHEMA/HOVON group have reported lower CR rates in 72 CD56^+^ patients compared to those 579 CD56^−^ patients [38]. We reasoned the differences observed in the studies might be derived from the number of enrolled cases. Thus, we reanalyzed the 530 patients from both the APL97 and APL204 studies, which gave similar results to those for the APL204 study. In the PETHEMA/HOVON group, patients with CD56^+^ APL also reported poorer ECOG PS scores and lower albumin levels compared with our patients [38]. The characteristics of patients enrolled in the study or undergoing the antileukemic regimen adopted in both studies might explain these differences.

Our study demonstrated that overexpression of CD56 was correlated with inferior RFS and higher CIR. CD56 was found to be an independent adverse prognostic factor for RFS by multivariate analysis. However, the direct or indirect molecular mechanisms to explain why CD56 expression in APL is associated with poorer prognosis are not well understood. Sobas et al., compared the five-year outcome with their previous study. CIR went up from 22% to 33% in CD56^+^ patients, but was unchanged in CD56^−^ patients. Relapse was more frequently observed in CD56^+^ patients compared to CD56^−^ ones in a long-term observation. In our study, however, late relapse three or more years after randomization did not occur in CD56^+^ patients, and thereafter, both CIR curves plateaued in parallel. The discrepancies might result from differences in patient background and variations in therapies.

In this study, CD56 expression was determined to be one of the prognostic factors in APL patients, especially those whose initial WBC counts were more than 3.0 × 10^9^/L. This observation might explain why the prognosis of patients with lower initial WBC counts was improved by ATRA plus chemotherapy [20]. Moreover, tamibarotene maintenance also improved prognosis [21,22]. Additional research is needed to ascertain the underlying reason for the poorer prognosis of CD56^+^ APL patients with higher initial WBC counts. A recent PETHEMA-LPA2012 study, which includes intensified consolidation for CD56+ group, will suggest the benefit of modification on the regimen with ATRA and chemotherapy.

The extramedullary relapse rate did not increase in our 530 patients enrolled in the APL97 and APL204 studies, while the PETHEMA/HOVON group and PETHEMA/HOVON/PALG/GATLA group have reported a higher risk of extramedullary relapse in their analysis of 651 and 956 patients, respectively [38]. This difference might be because our studies included prophylactic intrathecal injection after recovery from consolidation therapy.

In this study, overexpression of CD56 was not correlated to OS. The relapsed patients received tamibarotene, ATO and/or gemutuzumab ozogamicin as well as stem cell transplantation [21,22]. The reason why RFS and CIR were inferior in CD56^+^APL but not OS might be explained by the efficacy of salvage therapy with these drugs after recurrence of APL.

We think that CD56 is a next important prognostic factor to initial leukocyte count and maintenance in the treatment with ATRA and chemotherapy. It might be more important than other characteristics of APL cells, including secondary chromosomal abnormality, FLT3 mutations, multidrug resistant related factors, and BCR3 *PML-RARA* isoform. The clinical usage of CD56 expression in APL might be more important, if we assess quantitative change of CD56 over time by an advanced multicolor flow cytometry. Recently, in many institutes, we have evaluated clinical outcome of APL with the product of *PML-RARA*. However, we might need to redefine the role of multicolor flow cytometry during and after the treatment of APL as well as that adopted in the treatment of acute lymphoblastic leukemia.

Although this study has mainly focused on the clinical significance of CD56 in APL patients treated with ATRA plus chemotherapy regimen, we have also the results of treatment with ATO. Lou Y et al. [43] reported that overexpression of CD56 is a potentially unfavorable prognostic factor in 184 newly diagnosed APL patients treated with ATO-based frontline therapy. Recent studies suggest more successful outcomes can be achieved by using a combination of ATRA and ATO in patients with APL, especially for low- and intermediate-risk groups [44,45,46,47,48]. However, the clinical impact of CD56 was not clearly determined in these studies. The combination of ATRA and ATO could change the previous prognostic factors, including CD56, especially in the low-risk group. However, this combination therapy might have less impact in the high-risk APL group or for patients with recurrent disease. Accordingly, it is still important to determine prognostic factors such as overexpression of CD56 in APL patients, especially those with higher initial WBC counts.

## 5. Conclusions

CD56 has been continuously an independent unfavorable prognostic factor for RFS in APL patients treated with ATRA and chemotherapy followed by maintenance therapy.

## Figures and Tables

**Table 1:** Demographics and Clinical Characteristics.

Characteristic	Before Induction (*n* = 344)	Randomly Assigned for Maintenance Therapy		*p*-Value
		ATRA (*n* = 135)	Tamibarotene (*n* = 134)	
Age (years)				0.597
Median (range)	48 (15–70)	48 (15–70)	46 (16–69)	
Sex				0.758
Male/Female	183/161	70/65	72/62	
Performance status				0.858
0/1/2/3	188/126/19/11	72/50/8/5	78/43/8/5	
Leukocyte count (×10^9^/L)				0.841
Median (range)	1.4 (0.1–127)	1.3 (0.2–111)	1.4 (0.2–88.5)	
APL cell count (×10^9^/L)				0.502
Median (range)	0.3 (0–109)	0.2 (0–09)	0.4 (0–87)	
Platelet count (×10^9^/L)				0.343
Median (range)	31 (1–470)	28 (2–208)	32 (1–470)	
Sanz’s risk category				0.939
Low	117	47	44	
Intermediate	157	62	64	
High	70	26	26	
Fibrinogen level (mg/dL)				0.578
Median (range)	144 (8–810)	147 (27–593)	137 (8–496)	
FDP				0.784
Median (range)	50.3 (0–800)	53.7 (2.5–800)	51.4 (0–576.5)	
DIC score				0.946
0–2	37	14	15	
3–9	241	93	97	
Undetermined ^†^	66	28	22	
FAB subtype				0.434
M3/M3v	323/21	126/9	128/6	
Induction therapy group				0.984
A/B/C/D	112/48/70/114	47/18/26/44	45/20/26/43	
Additional Chromosome change other than t (15;17)				0.453
None	225	93	88	
Present	111	39	45	
Undetermined *	8	3	1	

FAB indicates French-American-British classification; ATRA indicates all-trans retinoic acid. * undetermined either because of insufficient sample or non-dividing cells; ^†^ undetermined because of insufficient sample.

**Table 2:** Risk factors for CR.

Clinical Features	Cases Achieving CR	Cases Who did not Achieve CR	*p*-Value
	No. of Cases	No. of Cases	
**Total**	319	25	
Age (years)			0.283
15–59	258	18	
60–70	61	7	
Sex			0.124
Male/Female	166/153	17/8	
FAB subtype			0.201
M3/M3v	301/18	22/3	
Leukocyte count (×10^9^/L)			0.011
<3.0	220	11	
≥3.0	99	14	
			0.044
<10.0	258	16	
≥10.0	61	9	
Platelet count (×10^9^/L)			0.244
<40.0	192	18	
≥40.0	127	7	
Sanz’s risk category			0.130
Low	110	7	
Intermediate	148	9	
High	61	9	
Performance status			0.397
0/1/2/3	178/113/18/10	10/13/1/1	
CD34			0.417
<10%	225	18	
≥10%	79	4	
CD56			0.212
<10%	263	17	
≥10%	40	5	
Additional Chromosome change other than t (15;17)			0.351
None	211	14	
Present	101	10	
Induction therapy			0.066
A/B/C/D	109/45/61/104	3/3/9/10	

Categorical data were compared using χ2-test and Fisher’s exact test for categorical variables. FAB indicates French-American-British classification.

**Table 3:** (**a**) Univariate analyses for RFS. (**b**) Multivariate analyses for RFS.

**(a)**					
**Clinical Features**	**No. of Cases**	**Median (Range)**	**HR**	**95% CI**	***p*-Value**
Age (years)					
15–59 vs. 60–70	221 vs. 48	46 (15–70)	0.62	0.21–1.78	0.373
Sex					
Male vs. Female	141 vs. 128		0.73	0.36–1.47	0.376
Leukocyte count (×10^9^/L)					
<3.0 vs. ≥3.0	184 vs. 85	1.3 (0.2–111)	2.72	1.36–5.45	0.003
<10.0 vs. ≥10.0	217 vs. 52		3.39	1.67–6.87	<0.001
Platelet (×10^9^/L)					
<40 vs. ≥40	164 vs. 105	30 (1–470)	0.77	0.37–1.59	0.477
Sanz’s risk category					
Low, Intermediate, and High	52, 126, and 91				0.001
Performance status					
0, 1, 2, and 3	149, 94, 16 and 10				0.302
CD34-positive blast					
<10% vs. ≥10%	191 vs. 65	3 (0–91)	2.13	1.02–4.45	0.040
CD56-positive blast					
<10% vs. ≥10%	221 vs. 34	2 (0–99)	3.04	1.34–6.90	0.005
Additional Chromosome change other than t (15;17)					
None vs. Presence	181 vs. 84		1.09	0.53–2.26	0.821
Induction Therapy					
A, B, C, and D	92, 38, 52 and 87				0.005
Maintenance Therapy					
ATRA vs. Tamibarotene	135 vs. 134		0.44	0.21–0.93	0.027
**(b)**					
**Clinical Features**	**No. of Cases**	**Median (Range)**	**HR**	**95% CI**	***p*-Value**
Leukocyte count (×10^9^/L)					
<10.0 vs. ≥10.0	204 vs. 51	1.4 (0.2–111)	3.55	1.68–7.50	0.001
CD56-positive blast					
<10% vs. ≥10%	221 vs. 34	2 (0–99)	3.19	1.40–7.27	0.006
Maintenance Therapy					
ATRA vs. Tamibarotene	125 vs. 130		0.41	0.19–0.91	0.028

(a) Statistical analyses were done by log-rank test. (b) Statistical analyses were done by Cox-proportional-hazards-model.

**Table 4:** CD phenotypes and clinical outcome.

CD No.	No. of Cases	OS (%)		*p*-Value	EFS (%)		*p*-Value	No. of Cases	RFS (%)		*p*-Value	CIR (%)		*p*-Value
		−	+		−	+			−	+		−	+	
CD2	193 vs. 107	90.5	81.9	0.084	84.3	72.7	0.030	153 vs. 80	92.2	83.8	0.053	6.7	16.6	0.019
CD4	223 vs. 42	87.2	97.6	0.116	78.7	95.2	0.029	170 vs. 37	87.6	94.6	0.204	12.1	2.7	0.094
CD5	246 vs. 11	88.4	100.0	0.231	80.3	90.0	0.366	192 vs. 10	87.5	90.0	0.847	11.7	11.1	0.932
CD7	299 vs. 15	88.4	80.0	0.094	81.8	66.7	0.033	236 vs. 10	89.8	80.0	0.372	10.0	10.0	0.979
CD8	243 vs. 10	88.6	100.0	0.259	80.9	88.9	0.445	187 vs. 9	88.8	88.9	0.970	10.4	12.5	0.873
CD11b	95 vs. 17	86.9	76.5	0.216	83.1	76.5	0.467	74 vs. 11	93.2	100.0	0.377	6.76	0.0	0.372
CD14	301 vs. 20	88.2	90.0	0.319	80.5	85.0	0.615	239 vs. 14	89.1	85.7	0.741	10.7	7.1	0.689
CD15	67 vs. 22	87.9	77.3	0.190	82.0	77.3	0.543	55 vs. 15	90.9	100.0	0.263	9.1	0.0	0.258
CD19	284 vs. 34	87.7	88.2	0.756	80.9	79.0	0.615	224 vs. 24	88.8	91.7	0.646	10.4	9.1	0.751
CD20	244 vs. 9	89.1	100.0	0.292	81.3	88.9	0.528	189 vs. 8	88.4	87.5	0.941	10.8	12.5	0.858
CD34	243 vs. 83	89.4	89.1	0.158	82.2	75.5	0.172	191 vs. 65	91.1	81.5	0.040	8.5	17.5	0.056
CD56	280 vs. 45	89.4	78.9	0.069	83.1	66.1	0.007	221 vs. 34	91.0	76.5	0.005	8.1	23.5	0.004
DR	245 vs. 72	87.9	88.8	0.732	80.2	81.8	0.937	191 vs. 57	89.9	87.0	0.850	6.8	5.3	0.997

OS, overall survival; EFS, event-free survival; RFS, relapse-free survival; CIR, cumulative incidence of relapse; DR, HLA-DR.

**Table 5:** Clinical feature of CD56^+^ and CD56^−^ patients.

Characteristic	CD56-Positive		CD56-Negative		*p*-Value
	(*n* = 45)		(*n* = 280)		
Age (years)					0.903
Median (range)	45 (20–69)		48 (15–70)		
Sex					0.068
Male/Female	18/27		153/127		
Performance status					0.363
0/1/2/3	21/21/3/0		154/102/14/10		
Leukocyte count (× 10^9^/L)					0.304
Median (range)	1.7 (0.4–27)		1.3 (0.1–111)		
APL cell count (× 10^9^/L)					0.543
Median (range)	0.7 (0–96.5)		0.2 (0–109)		
Platelet count (× 10^9^/L)					0.569
Median (range)	33 (3–160)		30 (1–237)		
Sanz’s risk category					0.939
Low	11		93		
Intermediate	21		132		
High	13		55		
Fibrinogen level (mg/dL)					0.478
Median (range)	166 (45–545)		139 (8–810)		
FDP					0.522
Median (range)	51.2 (7.5–255.5)		52.5 (0–800)		
DIC score					0.717
0–2	5		28		
3–9	30		203		
Undetermined ^†^	10		49		
Morphology					0.172
M3/M3v	40/5		264/16		
Induction therapy group					0.897
A/B/C/D	13/6/11/15		90/40/55/95		
Additional Chromosome change other than t (15;17)					0.923
None	30		184		
Present	15		89		
Undetermined *	0		7		

FAB indicates French-American-British classification; ATRA indicates all-*trans* retinoic acid. * undetermined because of insufficient sample or non-dividing cells; ^†^ undetermined because of insufficient sample.
